# Novel Platform for Quantitative Assessment of Functional Object Interactions After Stroke

**DOI:** 10.1109/TNSRE.2022.3226067

**Published:** 2023-02-01

**Authors:** Rashida Nayeem, Won Joon Sohn, Julie A. DiCarlo, Perman Gochyyev, David J. Lin, Member, IEEE

**Affiliations:** Department of Electrical and Computer Engineering, Northeastern University, Boston, MA 02115 USA.; Department of Electrical and Computer Engineering, Northeastern University, Boston, MA 02115 USA.; Center for Neurotechnology and Neurorecovery, Department of Neurology, Massachusetts General Hospital, Harvard Medical School, Boston, MA 02114 USA.; Center for Neurotechnology and Neurorecovery, Department of Neurology, Massachusetts General Hospital, Harvard Medical School, Boston, MA 02114 USA.; Center for Neurotechnology and Neurorecovery, Department of Neurology, Massachusetts General Hospital, Harvard Medical School, Boston, MA 02114 USA.; Department of Electrical and Computer Engineering and the Department of Biology and Physics, Northeastern University, Boston, MA 02115 USA.

**Keywords:** Index Terms—, Motor impairment assessment, stroke, quantification of functional skill, low-cost portable therapy device, complex-object manipulation, ecologically valid task

## Abstract

Many persons with stroke exhibit upper extremity motor impairments. These impairments often lead to dysfunction and affect performance in activities of daily living, where successful manipulation of objects is essential. Hence, understanding how upper extremity motor deficits manifest in functional interactions with objects is critical for rehabilitation. However, quantifying skill in these tasks has been a challenge. Traditional rehabilitation assessments require highly trained clinicians, are time-consuming, and yield subjective scores. This paper introduces a custom-designed device, the “MAGIC Table”, that can record real-time kinematics of persons with stroke during interaction with objects, specificallya ‘cup of coffee’. The task and its quantitative assessments were derived from previous basic-science studies. Six participants after stroke and six able-bodied participants moved a 3D-printed cup with a rolling ball inside, representing sloshing coffee, with 3 levels of difficulty. Movements were captured via a high-resolution camera above the table. Conventional kinematic metrics (movement time and smoothness) and novel kinematic metrics accountingfor object interaction(risk and predictability) evaluated performance. Expectedly, persons with stroke moved more slowly and less smoothly than able-bodied participants, in both simple reaches and during transport of the cup-and-ball system. However, the more sensitivemetric was mutual information, which captured the predictability of interactions, essential in cup transport as shown in previous theoretical research. Predictabilitysensitively measured differences in performance with increasing levels of difficulty. It also showed the best intraclass consistency, promising sensitive differentiation between different levels of impairment. This study highlights the feasibility of this new device and indicates that examining dynamic object interaction may provide valuable insights into upper extremity function after stroke useful for assessment and rehabilitation.

## I. INTRODUCTION

UPPER extremity (UE) motor impairment is common after stroke, affecting nearly two-thirds of the survivors [1]. Upper extremity motor deficits, such as muscle weakness, changes in muscle tone, and dexterity are a common cause of stroke-related disability [1], [2]. Current assessments of upper extremity impairments after stroke, such as the upper extremity Fugl-Meyer scale (UE-FMA) require a highly trained clinician to administer and their execution is time-consuming and subjective; in addition, the scales are prone to ceiling effects [3], [4], [5]. Tests that are specifically designed to assess functional abilities, such as the Wolf Motor Function Test (WMFT) or the Action Research Arm Test (ARAT), involve scores that rely on course-grained and subjective ratings [6], [7]. Although these tests serve as gold standards in clinical research, the scores do not adequately reflect the individual’s ability to engage in activities of daily living, such as performing hygiene or eating and drinking [3], [8]. Success in these essential tasks hinges on the ability to manipulate objects.

Many objects that individuals interact with in daily life have internal dynamics that must be managed for successful use [9], [10], [11]. Such interactions pose challenges to coordination that are absent in unconstrained movements which have been predominant foci of motor neuroscience research [12]. For example, when leading a cup of coffee or a glass of water to one’s mouth, the hand applies a force not only to the cup, but also indirectly to the liquid which acts back on the hand, potentially perturbing its trajectory [13]. The hand must make subtle adjustments to avoid spilling. Even though such interactive tasks are essential in daily life, it has been difficult to concisely measure performance [8].

**Fig. 1.**
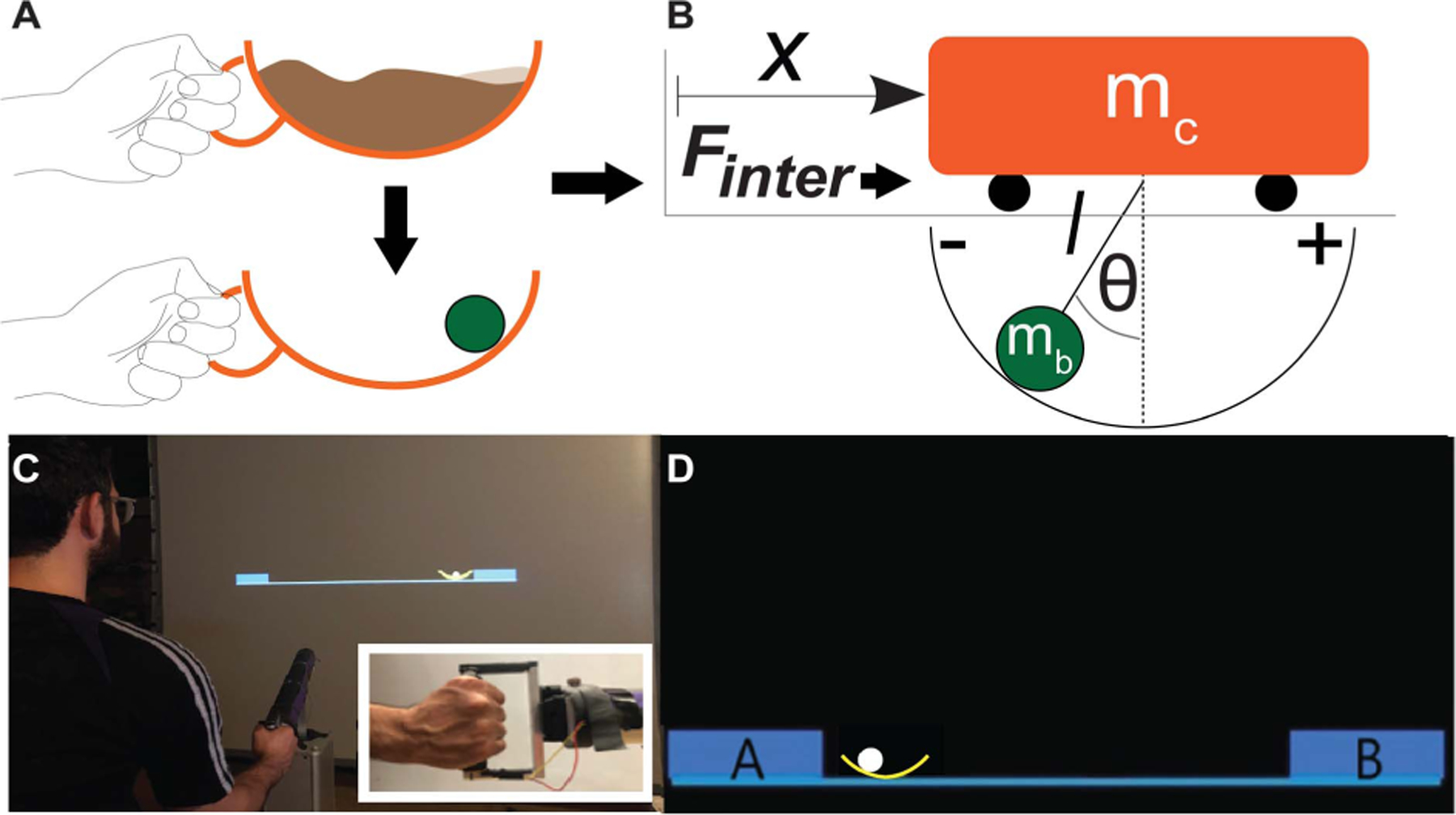
**A**. Hand grasping a cup of coffee. The cup of coffee was simplified to a two-dimensional arc with a rolling ball inside representing the sloshing coffee. **B**. Mechanical model of a cart with a suspended pendulum to represent the dynamics of the object. **C**. Implementation of the task in a virtual environment. Subjects controlled the position of the 2D cup via a robotic manipulandum, with the handle shown in the inset. The ball forces were transmitted back on their hand. **D**. Screen display with the cup and ball and the target boxes as participants see during the experiment.

Using the example of transporting a cup of coffee, Sternad and colleagues developed an experimental testbed that enabled quantitative measurements of interactions with an object that has nonlinear internal dynamics. The experimental paradigm simplified the task to moving a cup with a rolling ball inside (Fig.1A) [9], [14], [15]. This system was modeled with a known mechanical model, a cart with a suspended pendulum moving on a frictionless line (Fig.1B). By studying interaction with a known system, the analysis could focus on the object dynamics in the context of a task goal. This model system was first implemented in a virtual environment where the participant moved the cup-and-ball system via a robotic manipulandum that also transmitted the force of the ball to the hand (Fig.1C, D). The cup-and-ball system can display highly varying and potentially chaotic dynamics, resulting in complex interaction forces [9], [10]. Importantly, these nonlinear forces evolve rapidly such that corrections based on perceived errors are largely insufficient due to latencies in the sensorimotor loop [16]. Hence, humans need to learn to predict and preempt these forces. With the development of novel metrics, previous work found that neurotypical individuals aimed to make interactions with the cup-and-ball system more predictable, quantified by mutual information. Participants increased the mutual information about the object dynamics by exploiting resonance frequencies [15], [17], converging to the optimal phase of the system [9], stabilizing internal dynamics [11], [12], and appropriately initializing the object [10], [18]. Counter to common expectations, subjects did not minimize expended effort, nor increase the smoothness of the object kinematics [9], [10], [15]. Based on this rich set of insights into how able-bodied humans perform this task, the present study advanced this paradigm to a platform that can be used to quantitatively evaluate upper extremity deficits in persons with stroke [44].

**Fig. 2.**
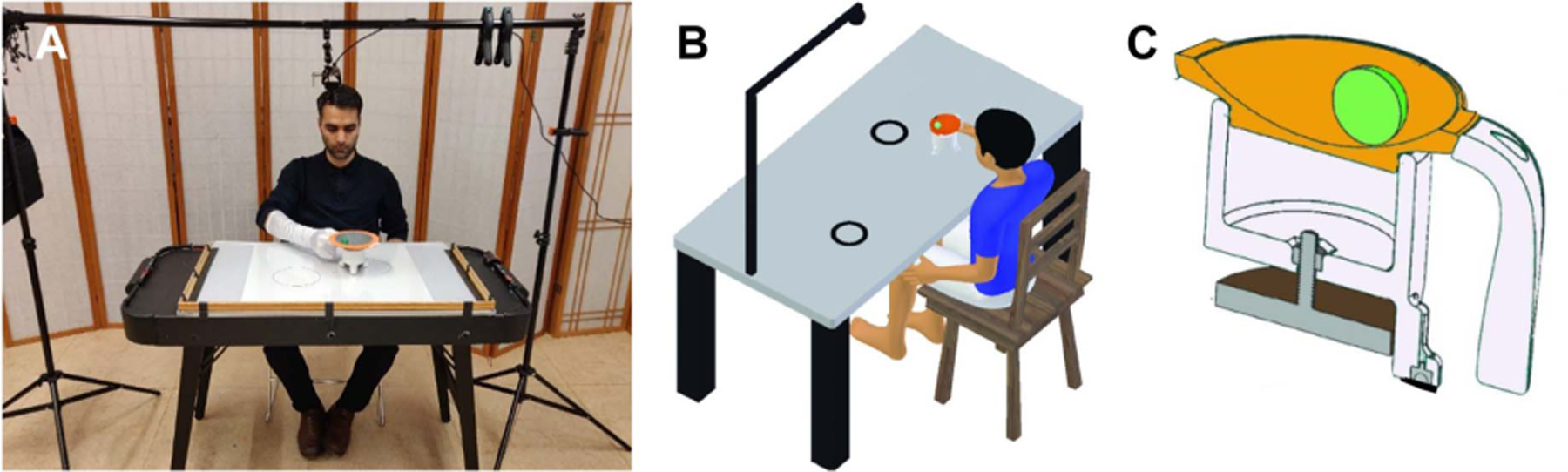
**A**. The MAGIC Table with the camera frame and webcam mounted above the table. **B**. Subject sits at a table with two circular targets drawn on the table surface. The subject moves the cup with the ball rolling inside from one target to the other. A web camera was mounted above the table surface to record the cup-and-ball kinematics in real time. Participants wear a white smooth glove to color-match the table surface and avoid errors in the color-based object detection. **C**. Cross-sectional view of the 3D-printed cup visualizes the magnet at the bottom of the cup and the metal green ball that rolls inside the orange bowl. The computer vision algorithm distinguishes objects by color to extract shapes and their position information.

To allow individuals with a wide range of motor capabilities to be measured, the virtual task was converted to a real three-dimensional task where subjects transported a cup with a ball rolling inside on a smooth table surface [19] (Fig.2). The MAGnetic Interactive and Creative Table, or MAGIC Table, consisted of a camera above the table connected to a mobile computer to track object kinematics in real time, eliminating the need for marker-based recording. This portable device was constructed out of low-cost and widely obtainable components. This study demonstrates the feasibility and efficacy of the MAGIC Table for assessment of upper extremity deficit in persons with stroke.

Our study design and analysis were guided by the following questions: 1) Do persons with upper extremity impairments after stroke exhibit differences when performing this functional task in comparison to able-bodied individuals? 2) Can we identify and quantify differences between the dominant upper extremity in able-bodied individuals and the “less affected” ipsilesional (same side as the lesioned brain hemisphere) side in persons with stroke? 3) Are our measures sensitive to identify differences between the ipsi- and contralesional (opposite side to the lesioned brain hemisphere) sides in persons with stroke? 4) Can our new task sensitively measure differences in performance with different levels of challenge? 5) Can our new metrics differentiate performance between persons with stroke that have distinct impairment levels? If successful, the MAGIC Table presents a paradigm-changing opportunity for rehabilitation: it allows quantitative assessment with theory-based metrics in a task reflective of self-feeding.

## II. METHODS

### A. Design of the MAGIC Table

The MAGIC Table consists of a large white board (60.9 × 91.4 cm) that is secured to a height-adjustable table (Fig.2A). Circles can be drawn on the whiteboard to serve as targets for movement tasks. Above the white board, a camera is mounted on an overhead frame (EMART 8.5 × 10 ft Photo Backdrop Stand), the camera was a variable focal-length webcam (RGB ELP 2.8–12 mm, Varifocal Lens HD 1080P Webcam, PN: ELP-USBFHD01MBFV). For testing in the hospital, a smaller whiteboard (43.18 × 58.42 cm) could be fit on top of a bedside table to enable participation by those that are bedridden. If less space is available, a tripod can be attached to the table with an adjustable clamp to position the camera above the board (Fig.2B). A previous study employed this set-up for collecting data from dystonic children who were in a hospital bed [19].

The cup was 3D-printed (using orange thread) to allow for customization of size and diameter. A 67 g steel ball, painted green, was placed inside the cup (BC Precision). The two colors were chosen to achieve maximum contrast for detecting and differentiating the ball from the cup background (Fig.2C). The cup was fixed to a 3D-printed base with an ergonomic handle. A height-adjustable magnet was screwed to the base of the cup to create attraction forces that prevented any tilting or raising of the cup, which would have distorted the kinematic recordings (McMaster PN: 7132T25, Diameter: 2.57 in). The distance between the magnet and the table could be adjusted to tune the magnetic strength to ensure that the cup glided easily across the surface while remaining in contact with the whiteboard. The legs at the bottom of the cup were covered with low-friction felt. The total cost to build the MAGIC Table was less than $200 (excluding the price of the computer for data acquisition). The itemized costs were the following: magnetic whiteboard ($30), RGB webcam ($55), and parts for the 3D-printed cup ($70).

**Fig. 3.**
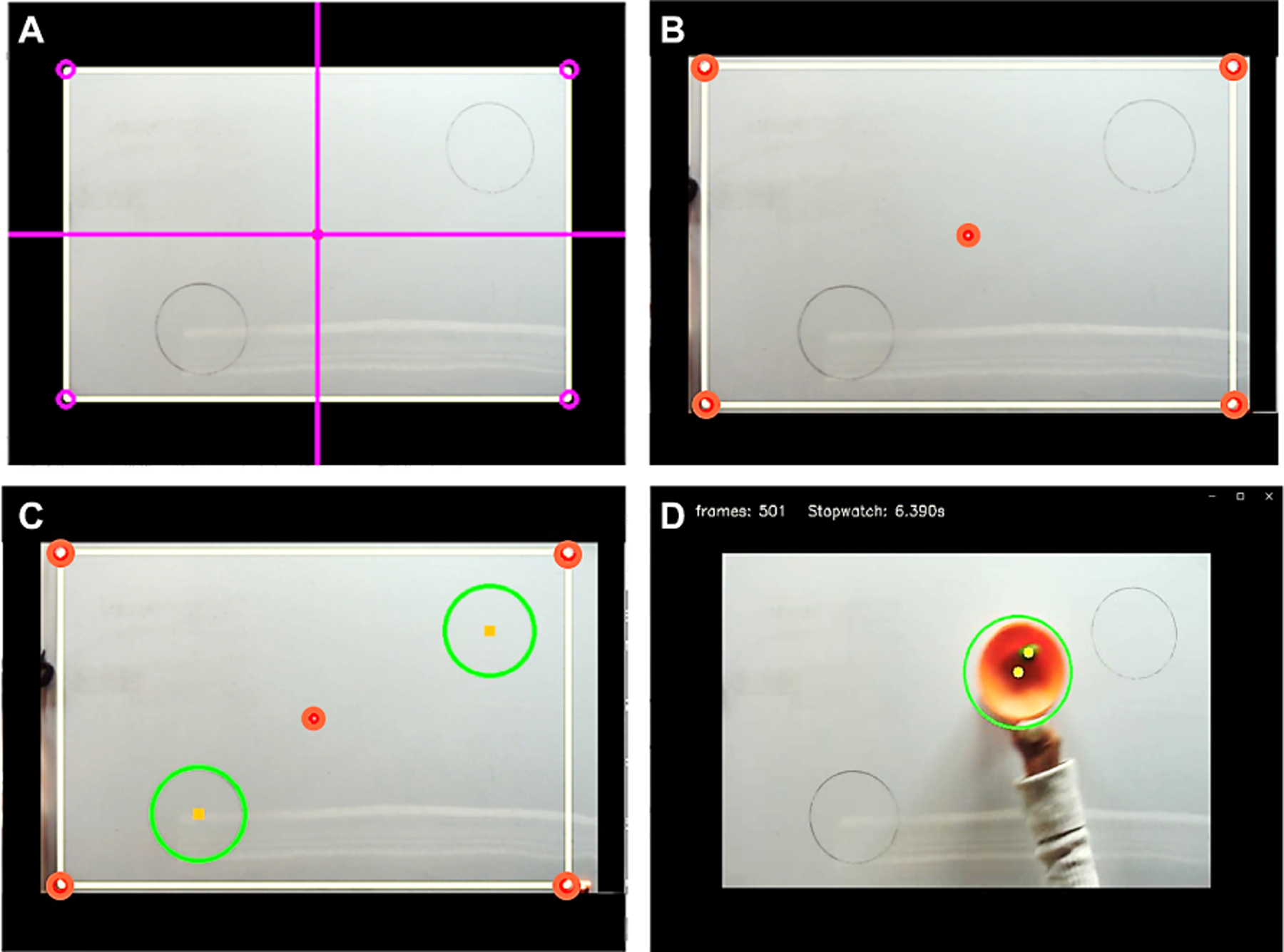
Stages of the experiment setup and execution as seen on the portable computer. **A**. View of finder screen with which the experimenter adjusts the camera position. **B**. Five-point matching procedure from the camera to the whiteboard. **C**. Detection of shapes by the color vision algorithm. **D**. Real-time video feed tracking the cup and the ball.

The USB 2.0 connector of the camera was plugged into a Microsoft Surface Pro 4 tablet PC (i5–6300U, 8GB RAM). The MAGIC Table algorithms for registration, target recognition, and task execution were written in Python and were run via one custom-developed program. Fig.3 shows the stages of setting up, calibrating, and executing the data acquisition. At the start of each experiment, the camera was adjusted to ensure the whiteboard was in view (Fig.3A). To keep the field-of-view consistent across trials, the camera was adjusted so that 5 points on a virtual overlay matched 5 fixed points on the whiteboard (Fig.3B). Prior to each trial the centers of the home and target circles were registered (Fig.3C). The position of the cup was tracked by the camera with a computer vision algorithm that recognized the center of the object in each frame (Fig.3D). The frame rate was 80 frames per second with a resolution of 640 × 480 pixels. More details of the algorithm can be found in [19]. The code for real-time object tracking in Python is provided in the online repository (https://github.com/wonjsohn/MAGIC_Table_basic).

### B. Experiment

#### 1) Participants:

The MAGIC Table was tested with six persons after stroke and six able-bodied participants in a feasibility experiment. Participant demographic and stroke characteristics are summarized in Table I. Participants after stroke were labeled as ‘PS’, and able-bodied participants were denoted as ‘AP’. PS were tested with both their contralesional (Contra) and and their ipsilesional (Ipsi) upper extremity. For characterizing impairment severity of PS, NIHSS scores were obtained within one month of the MAGIC Table testing. If persons with stroke had a Fugl-Meyer assessment within two weeks of their MAGIC Table experiment, their score was included. Data from AP participants show performance with their dominant upper extremity. All procedures were approved by the Northeastern University Institutional Review Board (IRB:18–08-01) and the Massachusetts General Hospital Review Board (IRB:2017P000868). All participants provided written informed consent.

### C. Movement Task and Experimental Design

Participants were instructed to slide the cup-and-ball on the whiteboard surface from a start circle to a target circle, and then back to the start circle, as fast as possible without losing the ball. The diameters of the two target circles were 14 cm with a distance of 52 cm between the centers of the two targets (see Fig.3). The movement was designed to allow individuals with different impairment levels to perform the task. In the starting position, the elbow was flexed and the cup rested on the start circle; the ball was also at rest. The movement began with an elbow extension towards the target circle, followed by a flexion back to the start circle. When the participant executed the task with their right upper extremity, the start and target circles were placed in the southwest and northeast corners of the board, respectively. When the participant executed the task with their left upper extremity, the positions of the start and target circles were mirrored to elicit the same extension-flexion movement with their arm. If the PS had issues reaching the target circle, they were encouraged to extend their upper extremity as far as they could. During the movement the upper extremity was not permitted to rest on the table surface. This required subjects to actively abduct their shoulder, and then extend and flex their shoulder and elbow joints during movement. Note, minimal wrist or hand movements were needed to complete the task as the participants held the cup handle with a comfortable grip. Subjects wore a white glove to avoid interference of skin color with the color-based detection of the cup and ball.

**Fig. 4.**
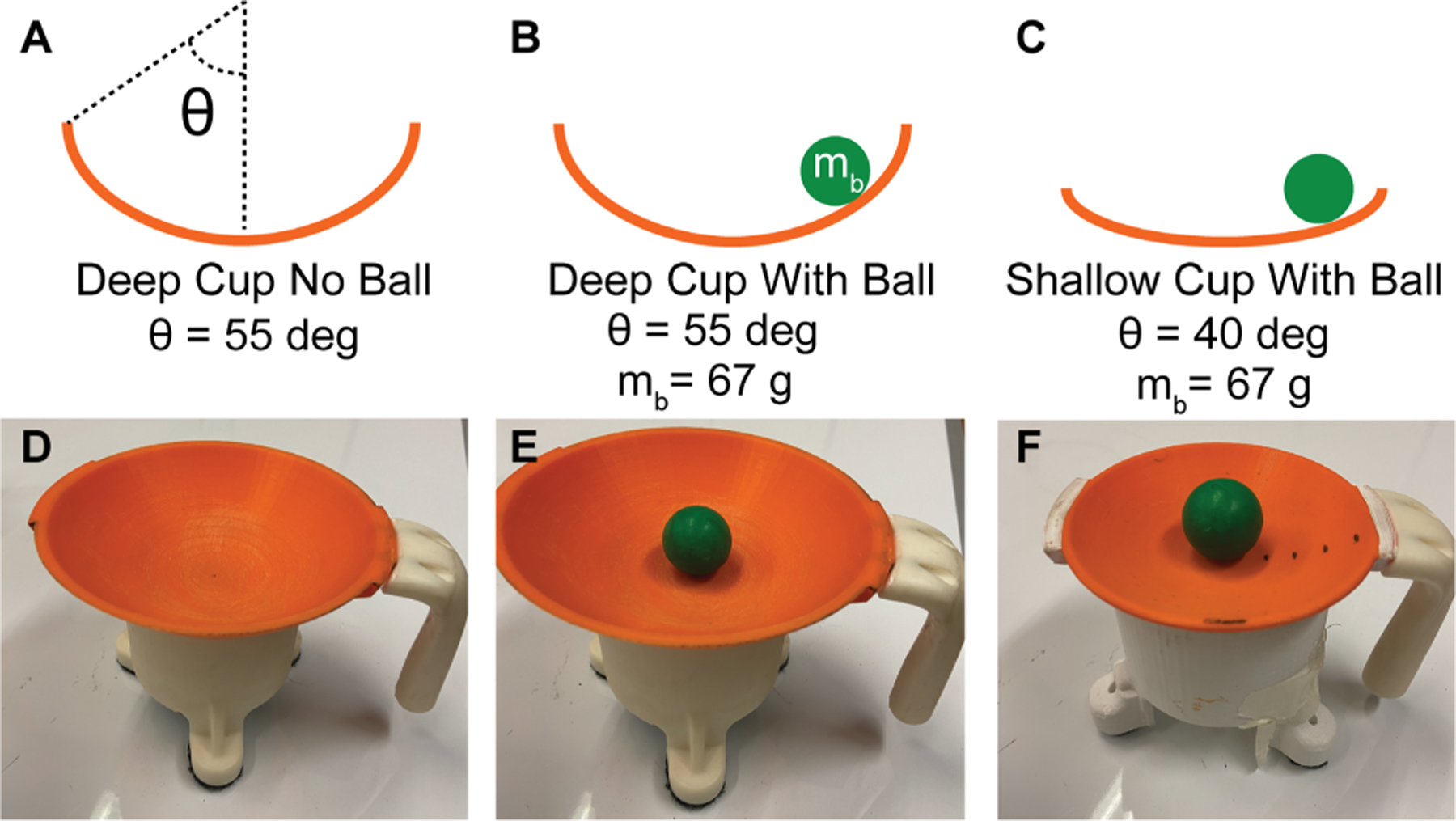
Model sketches and 3D-printed rendering of the cup and ball with different dimensions (radius of curvature and rim height); *θ* indicates the angle of the rim height. **A**. Model of the Deep Cup No Ball. **B**. Model of the Deep Cup With Ball. **C**. Model of the Shallow Cup With Ball. **D**. Real 3D-printed Deep Cup No Ball. **E**. Real 3D-printed Deep Cup With Ball. **F**. Real 3D-printed Shallow Cup With Ball.

The difficulty of the task was manipulated by using cups of different dimensions, i.e., different radius of curvature, and different rim heights. Fig.4 shows three levels of difficulty that were employed in this study. The easiest level was the Deep Cup No Ball, because this object presented no additional dynamics and essentially reduced the task to a simple reach with a mass added to the hand. The cup radius of curvature was 75 mm and its rim height was 55 deg, although radius and rim height were irrelevant for this condition. The second level of difficulty was the Deep Cup With Ball. The cup radius and the rim angle were the same as before, but with the ball added there was the risk of losing the ball if its angle exceeded 55 deg. The ball had a mass of 67 g and a radius of 1.1 cm. The third level of difficulty was the Shallow Cup With Ball. The cup radius was again 75 mm, but the cup had a rim height of only 40 deg, making it easier for the ball to escape. The ball had the same mass and radius.

Participants were seated at the long side of the MAGIC Table instructed to move the cup upon an auditory “Go” signal from the computer. When the cup returned to the start circle, another bell sound was triggered to signal the successful finish of the trial. Recordings for each trial ended when the center of the cup had been inside the start circle for 3 s. Each participant performed 20 trials for each condition in blocked fashion. The six PS completed each level of difficulty in the order of increasing difficulty with their ipsilesional side to gain familiarity with the task, then with their contralesional side. The six AP completed each level of difficulty in the same ascending order with their dominant side first, then with their non-dominant side. The experiment comprised a total of 120 trials and the experimental session lasted approximately 1 hour including the set up and the calibration.

#### 1) Model of the Object and the Task:

Even though the real cup was a 3D object, the instructed cup movements were along a line from the start to the target circle; hence the movements were confined to one dimension. Therefore, the cup and ball kinematics could be modeled using the same 2D cart-and-pendulum model that was used in previous research, Fig.1 [9], [10], [15]. Given that the angular movements of the ball were confined to the direction of movement, the pendulum had only one angular degree of freedom, and the following equations of motion described the system:

(1) $$\left(m_{c}+m_{b}\right)\ddot{X}=\underset{F_{ball}}{\underset{︸}{m_{b}l\left[{\dot{\theta }}^{2}sin\theta -\ddot{\theta }cos\theta \right]+F_{inter}}}$$

(2) $$\ddot{\theta }=-\frac{\ddot{X}}{l}cos\theta -\frac{g}{l}sin\theta .$$

*X* was the cup position, *θ* was the ball angle; the downward vertical orientation of the pendulum/ball defined 0 deg, with counterclockwise rotations denoted as positive. The mass of the cup was denoted by *m*_*c*_, the mass of the ball by *m*_*b*_, and the length of the pendulum was *l*. *F*_*inter*_ was the interaction force applied by the participant onto the cup, and *F*_*ball*_ was the force applied by the ball onto the cup.

### D. Data Processing and Definition of Metrics

The data was low-pass filtered with a 12 Hz cut-off frequency using an 8th-order Butterworth filter. The kinematic data was then linearly transposed such that the x-axis was along the movement direction (the line between the center of the start circle to the center of the target circle). The kinematics along this dimension were analyzed using the metrics described next. The data lent themselves for several conventional and novel metrics to quantify performance in this interactive task. All processing and analyses were performed in MATLAB.

#### 1) Movement Time:

Previous work demonstrated that persons with upper extremity deficits after stroke required more time to execute a reaching task and the duration correlated with functional scores [20], [21]. Hence, as a baseline measure, movement time with the rigid object was compared to that with the cup-and-ball object, with the hypothesis that movement time slows down with the increased coordinative challenge. Movement time comprised the time required to complete both extension and flexion. The start and end of the movement was defined at the moment when cup velocity in the direction of movement was above a specified threshold. This threshold was defined by the absolute cup velocity exceeding 5% of the maximum velocity in the same trial.

#### 2) Smoothness:

Smooth movements express the continuity of a movement and metrics of smoothness have been considered markers of skilled human motor behavior [22], [23]. Persons with stroke have been shown to exhibit intermittent, i.e. less smooth velocity profiles in their upper-limb movements, but their movements could become smoother during recovery [24], [25], [26]. Due to the object-hand interactions, we expect less smooth movements in the cup-and-ball task. Several different metrics have been developed to quantify smoothness of upper-limb movement, the most commonly used is mean squared jerk (the derivative of acceleration) [27]. However, this metric was shown to be sensitive to noise and provided inconsistent results for movement in persons with stroke [23], [28], [29]. The Spectral Arc (SPARC) method proved to be more reliable in quantifying smoothness of movement [23], [28], [29]. This metric estimated smoothness in the frequency domain, making the values independent of movement duration and amplitude.

The SPARC method applied a Fourier transform to the speed profile of the cup movement, *V* (*t*)*, $t\in \left[0,\;\;T\right]$*, to yield the frequency magnitude spectrum *V (ω)*. The Fourier spectrum was then normalized with respect to the DC-component, *V* (0), providing $\hat{V}\left(\omega \right)$; therefore, the smoothness values were unitless (arbitrary units, a.u.) [23]. Then, the arc length of the normalized Fourier spectrum was computed:

(3) $$SPARC=-{\int_{0}^{w_{c}}\left[{\left(\frac{1}{w_{c}}\right)}^{2}+{\left(\frac{d\hat{V}\left(\omega \right)}{d\omega }\right)}^{2}\right]}^{\frac{1}{2}}d\omega$$

(4) $$\hat{V}\left(\omega \right)=\frac{V\left(\omega \right)}{V\left(0\right.})$$

(5) $$w_{c}=min\left[w_{c}^{max},min\left[\omega ,\hat{V}\left(r\right)<\bar{V}\vee r>\omega \right]\right]$$

Less smooth movements have more frequency components resulting in a more complex envelope with a longer arc length. *ω*_*c*_ defined the upper limit of the frequency window that the arc length was calculated over; it was defined by the threshold $\bar{V}$ and was upper-bounded by$\omega_{c}^{max}$. This limit served to remove high-frequency noise from the signal. The current calculation adopted the recommended parameters given in [23]: $\bar{V}=0.05$ and $\omega_{c}^{max}=20\pi$ rad/s. A higher value of $\bar{V}$ would exclude critical features of the spectrum. Smoothness was calculated over the trial segment in which the absolute cup velocity was greater than 5% of the absolute peak velocity in the given trial.

#### 3) Risk:

In self-feeding activities humans seek to avoid the risk of spilling their drink or food. Hence, risk or safety margins have been evaluated in previous studies as indicator of successful completion of a task. During transport of the cup-and-ball, neurotypical young adults decreased risk of ‘spilling’ the ball when unconstrained by time [30]. In older individuals this risk of ‘spilling’ was greater, but it decreased with practice [31]. In this study we expected persons with stroke to show higher risk than able-bodied individuals.

To quantify how subjects reduced the chance of losing the ball, an energy margin was defined [10]. This metric used the energy of the ball to calculate the risk or likelihood of the ball to escape the cup. For the ball to remain in the cup, the total energy of the ball *E*_*Total*_, the sum of potential energy *E*_*Potential*_ and kinetic energy *E*_*Kinetic*_, cannot exceed a threshold. This threshold, referred to as escape energy *E*_*ESC*_, and was determined by the cup’s rim angle, cup radius, ball mass and gravity. If *E*_*Total*_ became greater than *E*_*ESC*_, the ball would escape from the cup, unless the energy was quickly dissipated. The safety or energy margin was the difference between *E*_*ESC*_ and *E*_*Total*_ at any given time point:

(6) $$E_{Kinetic}\left(t\right)=\frac{\dot{\theta }{\left(t\right)}^{2}l^{2}m_{b}}{2}$$

(7) $$E_{Potential}\left(t\right)=m_{b}gl\left(1-\text{cos}\;\theta \right)$$

(8) $$E_{Total}\left(t\right)=E_{Kinetic}\left(t\right)+E_{Potential}\left(t\right)$$

(9) $$E_{ESC}=m_{b}gl\left(1-\text{cos}\;\theta_{ESC}\right)$$

(10) $$E_{MARGIN}\left(t\right)=E_{ESC}-E_{Total}\left(t\right)$$

These calculations provided a time series of *E*_*MARGIN*_ over the course of the trial. The time points *t*_*i*_ of the local ball angle maxima, i.e., the moments when the ball angle magnitude was higher than at the adjacent time points, were determined. The mean of the *E*_*MARGIN*_ (*t*) at these moments was calculated and transformed into a risk metric by the following conversion:

(11) $$Risk=1-\frac{1}{n}\;\frac{1}{E_{ESC}}\sum_{i=1}^{n}E_{MARGIN}\left(t_{i}\right)$$

#### 4) Mutual Information:

As reviewed above, previous research demonstrated that predictability of object interactions was paramount and able-bodied subjects increased predictability with practice. We expected that individuals with stroke achieve lower predictability than able-bodied individuals. To quantify predictability of the object, mutual information (*MI*) was computed. This measure quantified how much the information about one variable predicted another variable; high *MI* conveys high predictability [32]. *MI* can assess both linear and nonlinear dependencies, making it a more robust measure than cross-correlation [32]. Specifically, *MI* computed the degree to which the cup kinematics could predict the temporal evolution of the ball dynamics. Since the cup and ball trajectories were close to sinusoidal, the kinematics were best represented by phase in state space. *MI* between cup phase *φ*_*cup*_ and ball phase *φ*_*ball*_ quantified the degree of predictability between the cup that the subject moved and the ball that was moved indirectly.

(12) $$\phi_{cup}\left(t\right)=\tan^{-1}\left[\frac{\dot{X}}{2\pi fX}\right],\;\;\;\;\;\;\phi_{ball}\left(t\right)=\tan^{-1}\left[\frac{\dot{\theta }}{2\pi f\theta }\right]$$

*MI* was then defined as:

(13) $$MI\left(\phi_{ball},\phi_{cup}\right)=\iint P\left(\phi_{ball},\phi_{cup}\right)ln\left[\frac{P\left(\phi_{ball},\phi_{cup}\right)}{P\left(\phi_{ball}\right)P\left(\phi_{cup}\right)}\right]d\phi_{ball}d\phi_{cup}.$$

*P* denotes the probability density functions for *φ*_*ball*_ and *φ*_*cup*_ computed using the distribution of the experimental data with histogram estimators [33]. *MI* is a dimensionless quantity represented on a natural log scale (nat) and was calculated over the trial segment where absolute cup velocity was greater than 5% of the peak velocity for a given trial.

### E. Statistical Analyses

Data were collected from the contralesional and ipsilesional upper extremity of six participants after stroke, and from both upper extremities of six able-bodied participants. For the sake of focus, the non-dominant upper extremity of able-bodied individuals was not included in the analysis. Hence, the data comprised six able-bodied subjects with 60 trials each, and six persons with stroke with, in principle, 120 trials each. However, two of the PS were collected at the hospital and due to time constraints PS1 and PS5 could only complete 25 and 43 trials, respectively.

Each of the four metrics was evaluated using a mixed-effects model, specifically a linear random-intercept model, with group (Contra, Ipsi and Able-Bodied) and level of difficulty as fixed effects and subject-specific intercepts as random effects. All trials of each subject were entered into the model. This analysis addressed whether persons after stroke exhibited differences in performance compared to able-bodied individuals in their contralesional and their ipsilesional side (question 1 and 2). The same model also examined whether the contralesional side differed from the ipsilesional side in persons with stroke (question 3). We also tested whether performance in all participants was affected by the different levels of difficulty (question 4).

With the level of power set to 0.80 and significance set to 0.05, the statistical tests were sufficiently powered to detect an effect size *d* of 0.36 or higher. The effect sizes were reported for differences that were statistically significant. To further probe whether our new metrics could reveal reliable differences in performance in PS with distinct impairment scores (question 5), we also computed intra-class correlation coefficients (ICCs). Using the mixed-effects model, ICCs quantified the sources of variance across participants after stroke. Low ICC values imply that most of the variance in the data was due to within-subject variance; high ICC values indicate a higher proportion of between-subject variance (showing greater between-subject differences and within-subject homogeneity), signifying that a metric is relatively consistent for a given participant. Two different types of ICC were computed: conditional ICC (from a model containing the two covariates group and difficulty) and unconditional ICC (from a model not containing any covariates). All statistical analyses were conducted using STATA 17.

## III. RESULTS

**Fig. 5.**
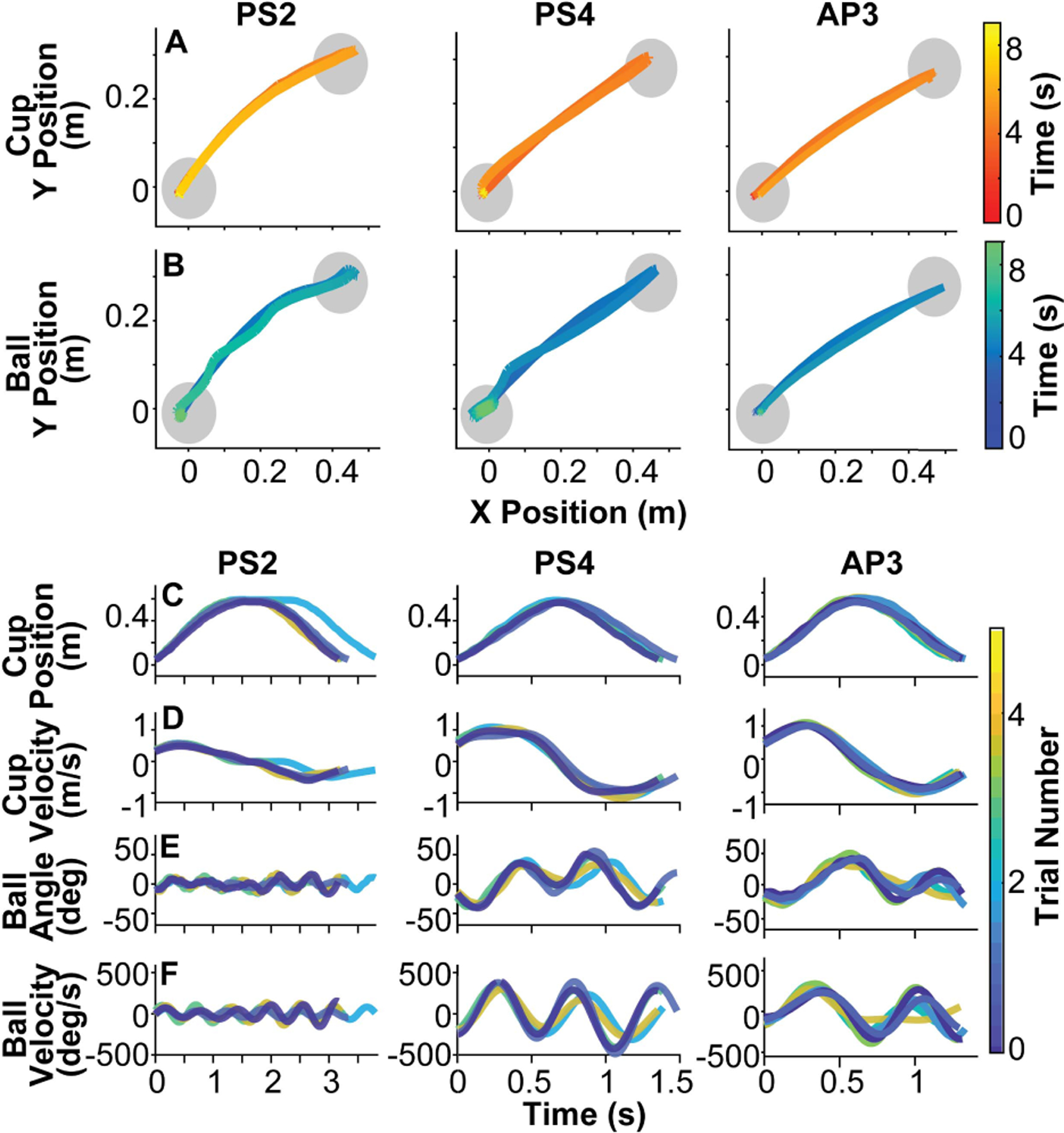
Exemplary kinematics of three participants, two participants after stroke (PS2 and PS4) and one able-bodied participant (AP3). **A.** Overhead view of displacements of the cup in the three participants. **B.** Overhead view of displacements of the ball in the three participants. In all panels the color gradients display the time elapsed as participants completed the task. The gray circles indicate the location and size of the start and target circles. The start and target circles are in the southwest and northeast corner from the participant’s perspective. PS2: Participant with apparent upper extremity weakness after stroke. PS4: Participant with no apparent upper extremity weakness after stroke. AP3: Able-bodied participant. **C**. Time series of the cup position. **D**. Time series of the cup velocity. **E**. Time series of the ball angle. **F**. Time series of the ball velocity. All time series were from the Deep Cup With Ball condition and measured along the axis of movement.

Fig.5A, B presents the overhead kinematics of the cup and the ball collected from two participants after stroke (PS2 and PS4) and one able-bodied participant (AP3). Fig.5C, D, E, F show the time series of cup and ball displacements and velocities in the direction of movement (defined between the centers of the start and target circles). Note the different scales on the time axes. The kinematics of each subject differed in the ball’s displacement and velocity, sensitively revealing different strategies for each performer.

### 1) Movement Time:

**Fig. 6.**
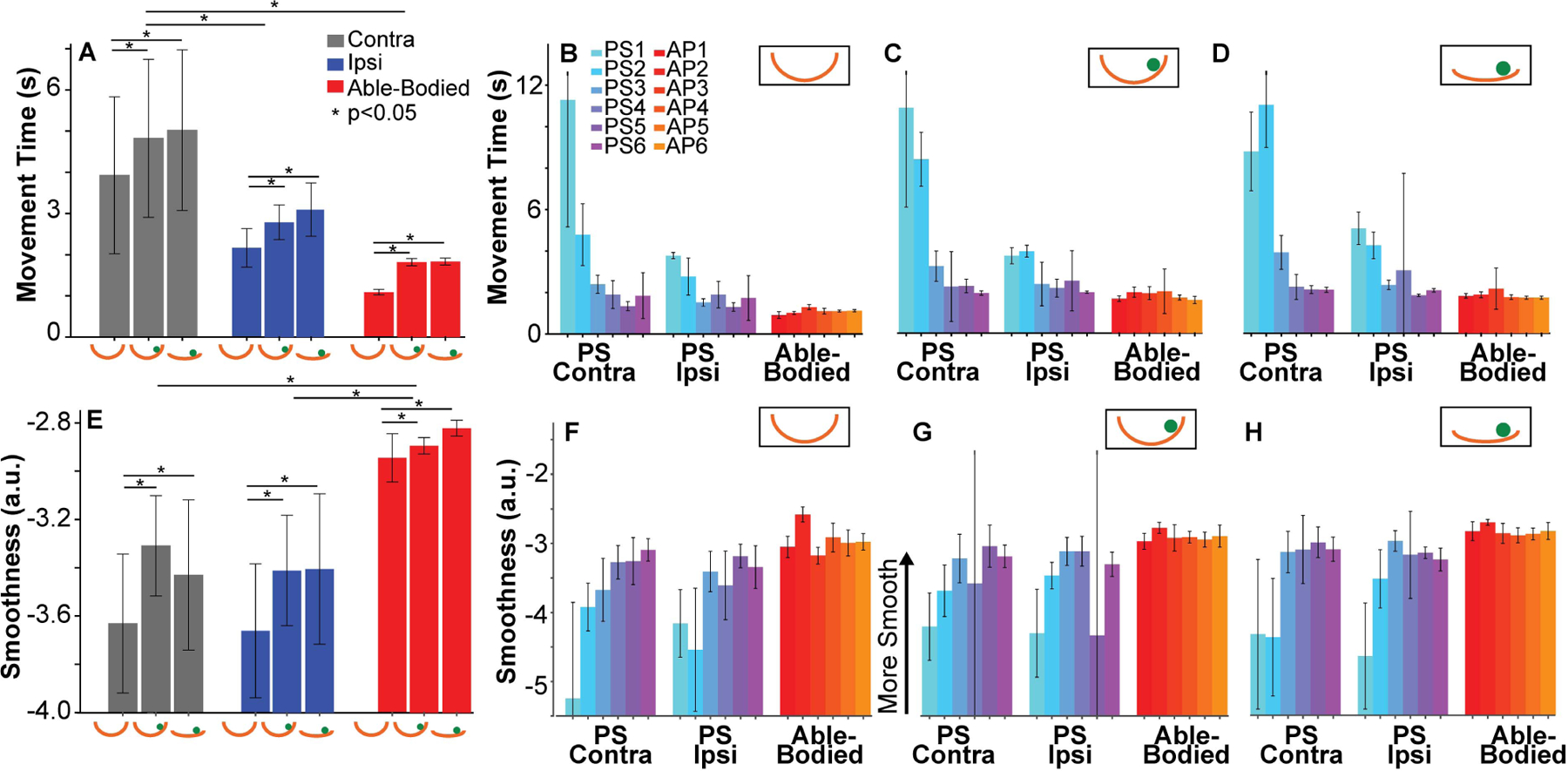
(*Top Row*) Performance measured by movement time. **A**. Movement times averaged across subject means for the contralesional (Contra) side of participants after stroke, ipsilesional (Ipsi) side of participants after stroke, and dominant side of able-bodied participants (Able-Bodied). Each bar represents the average and standard deviation of the group data. The bars represent Deep Cup No Ball, Deep Cup With Ball and Shallow Cup With Ball conditions. **B**-**D** Each bar displays the mean movement time for each participant, showing their average and standard deviation across all trials. **B**. Deep Cup No Ball condition. **C**. Deep Cup With Ball condition. **D**. Shallow Cup With Ball condition. (*Bottom Row*) Smoothness values measured with by the SPARC metric. Less negative or smaller values indicate that trajectories were smoother. **E**. Smoothness values averaged across subjects for upper extremity group (Contra, Ipsi, Able-Bodied). Performance is separated for each level of difficulty. **F**-**H**. Average and standard deviation of smoothness of each participant. **F**. Deep Cup No Ball condition. **G**. Deep Cup With Ball condition. **H**. Shallow Cup With Ball condition.

Fig.6A summarizes movement time averaged across subjects for each upper extremity (Contra, Ipsi, Able-Bodied) and for each level of difficulty (different cups with and without the ball). As expected, movement time of the PS contralesional arm was significantly slower than movement times of the AP dominant upper extremity (*p =* 0.006, *d =* 0.61). While movement time of PS’s ipsilesional arm did not differ from the dominant upper extremity of AP (*p =* 0.13), it was significantly faster than the contralesional side (*p <* 0.001, *d =* 0.26). Comparison across the different levels of difficulty showed that, when controlling for hand group, the average movement times in the easiest condition, Deep Cup No Ball, were significantly shorter than movement times in the two harder conditions, Deep Cup With Ball (*p <* 0.001, *d =* 0.17) and Shallow Cup With Ball (*p <* 0.001, *d =* 0.21). However, the two latter conditions were not different (*p =* 0.08). This confirmed that adding internal dynamics to the object indeed slowed the speed of the transport as expected. Fig.6B, C, D show the means and standard deviations of movement time for each subject split by the hand group and the three levels of difficulty. Each color represents a different subject. To evaluate whether movement time was consistent for a given PS, intraclass coefficients ICC were calculated. The unconditional and conditional ICC measures among the PS were at 0.57 and 0.61, respectively, indicating relatively high between-subject differences with more homogeneity within subjects.

### 2) Smoothness:

Fig.6E overviews the averages and standard deviations of smoothness for each of the three hand groups and difficulty levels. Note that less negative SPARC values indicate smoother trajectories. As expected, when controlling for difficulty, cup kinematics with the contralesional were significantly less smooth than those with the able-bodied arm (*p =* 0.005, *d =* 0.62); similarly, the ipsilesional arm was less smooth than the upper extremity of AP (*p =* 0.004, *d =* 0.59). However, smoothness values did not differentiate between PS’s contra- and ipsilesional upper extremity (*p =* 0.55), suggesting less sensitivity than movement time. Controlling for group, performance with the Deep Cup No Ball was less smooth than performance with the Deep Cup With Ball (*p <* 0.001, *d* = 0.19) and the Shallow Cup With Ball (*p <* 0.001, *d* = 0.22). This appeared counterintuitive, but reflected the additional demands when a moving ball was involved. The smoothness metric could not differentiate between the two conditions with the ball (*p =* 0.21). Fig.6F, G, H show average smoothness values of each subject for each hand group and level of difficulty. The unconditional and conditional ICC values in PS were estimated at 0.65 and 0.67, respectively, demonstrating that smoothness values attained by each participant were relatively consistent, i.e., there was higher correlation within subjects than between subjects.

### 3) Risk:

**Fig. 7.**
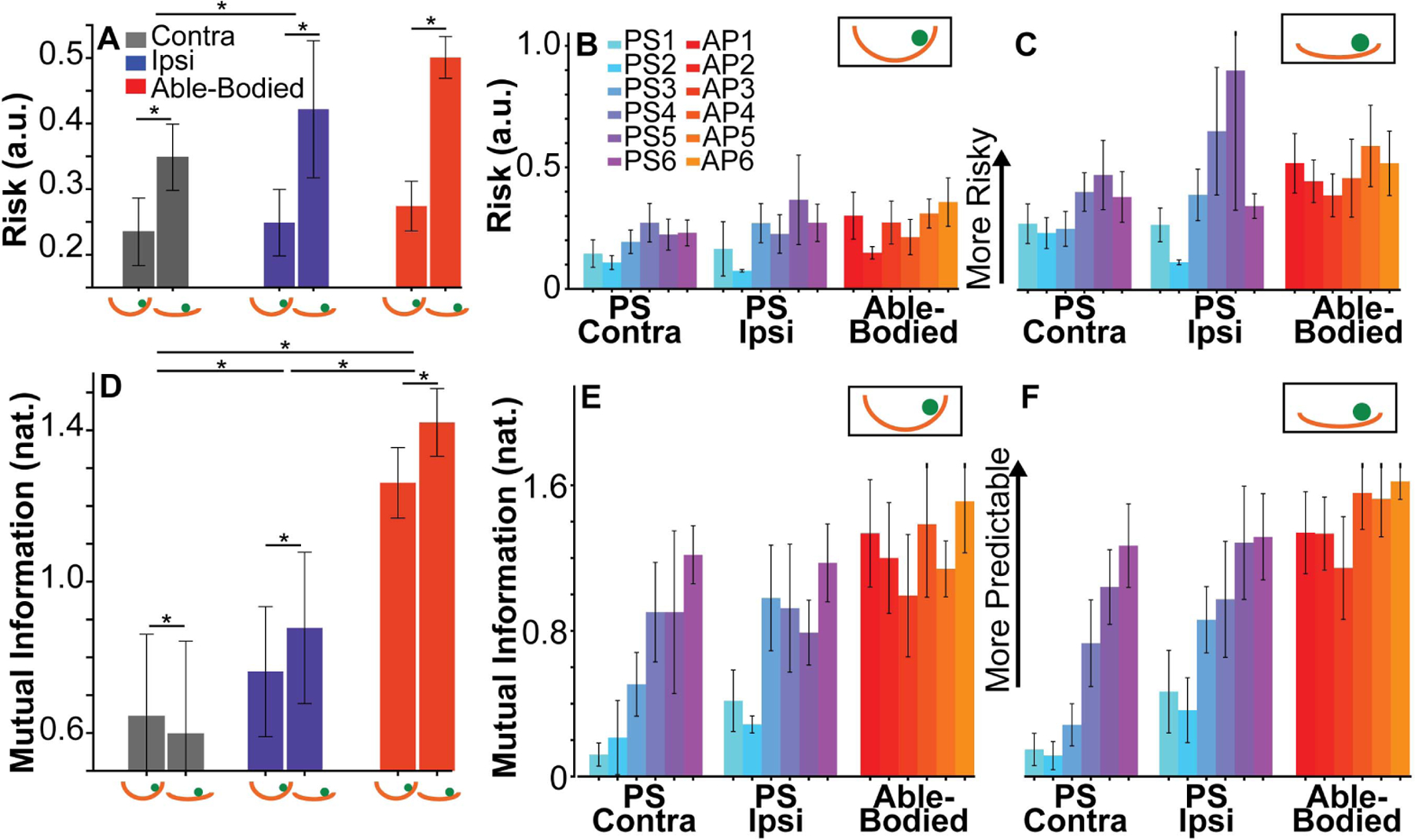
(*Top Row*) Performance measured by the risk metric. **A**. Average and standard deviation of risk across subjects in each group, Contra, Ipsi and Able-Bodied. The two bars for each group represent performance in Deep Cup With Ball and Shallow Cup With Ball conditions. **B**-**C**. Each bar shows the mean and standard deviation of risk for all trials of each participant in each group (Contra, Ipsi and Able-Bodied). Risk values for **B**. Deep Cup With Ball condition. **C**. Shallow Cup With Ball condition. (*Bottom Row*) Performance measured by mutual information between cup and ball kinematics as a metric for predictability. **D**. Average and standard deviations of mutual information for all participants of a hand group across Deep Cup With Ball and Shallow Cup With Ball conditions. **E**-**F**. Each bar shows the mean and standard deviation of mutual information from all trials of each participant. Data are separated by Contra, Ipsi and Able-Bodied. **E**. Deep Cup With Ball condition. **F**. Shallow Cup With Ball condition.

Fig.7A shows the averages and standard deviations of risk values across the three upper extremity levels, split by difficulty. Note that risk could only be calculated when the cup had a ball inside. Contrary to expectations, risk values of the PS group did not differ from those of the AP, neither in the ipsilesional extremity (*p =* 0.44), nor in the contralesional extremity (*p =* 0.08). Yet, risk in the ipsilesional upper extremity was significantly higher than in the contralesional upper extremity group (*p =* 0.001, *d =* 0.13). When averaging over group, risk in Deep Cup With Ball was significantly lower than in the Shallow Cup With Ball (*p <* 0.001, *d =* 0.47), as with a shallower cup it was easier to lose the ball. Fig.7B, C show risk values for each subject and difficulty level. Unconditional and conditional ICC values in persons with stroke were 0.31 and 0.38, respectively, indicating that there was more within-subject variability, i.e., the metric was not as discerning of different impairment levels as other metrics.

### 4) Mutual Information:

Average and standard deviation of mutual information values *MI* across subjects for each group and level of difficulty are summarized in Fig.7D. This metric could only be calculated when a ball was in the cup. High *MI* indicates that the ball kinematics were more predictable for the given cup kinematics. As expected, participants with stroke had lower *MI* values than able-bodied participants. *MI* of the contralesional upper extremity in PS was significantly lower than that in able-bodied individuals (*p <* 0.001, *d =* 0.75). In addition, the ipsilesional extremity of persons with stroke also had lower *MI* than the able-bodied individuals (*p =* 0.004, *d =* 0.51). Further, *MI* detected a significantly higher value in the ipsilesional arm compared to the contralesional arm (*p <* 0.001, *d =* 0.20). With these differences, *MI* not only distinguished between performance in AP versus PS, but also differentiated between the “affected” and “unaffected” side in PS. When comparing the two levels of difficulty, *MI* was significantly higher in the more difficult Shallow Cup With Ball condition (*p =* 0.01, *d =* 0.06). Overall, mutual information was the only metric that differentiated between all levels of group and difficulty. Fig.7E, F presents the subject averages of each participant split by hand group and difficulty. Unconditional and conditional ICC values in persons with stroke were estimated at 0.67 and 0.69, respectively. These were the highest ICC values across all metrics, demonstrating that *MI* was consistent within a subject while displaying larger differences between subjects.

## IV. DISCUSSION

This study presented a novel testing device that was developed on the basis of a theoretically grounded paradigm for assessing hand-object interactions, and evaluated its translation into a clinical context. Specifically, goal-oriented interaction with a complex object, core to numerous daily activities from stirring soup to drinking a cup of coffee, motivated an experimental platform for the assessment of upper extremity functional skill after stroke. Prior fundamental research developed the model-based task, tested it in a virtual implementation and validated novel metrics that quantified features of successful performance. This paradigm was converted into a real-world 3-dimensional task and a portable device was custom-designed for easy use in clinical settings. The MAGIC Table is an affordable and inclusive platform that provides precise kinematic data for model-based data analyses of functional interactions. Its practical and low-cost features may also allow use in rehabilitative assessment in the future.

To demonstrate its feasibility, this study compared task performance of six participants after stroke and six able-bodied participants using both conventional and novel kinematic metrics. Using the conventional metrics of movement time and smoothness, results showed that participants after stroke took longer to complete the task and exhibited reduced smoothness in comparison to able-bodied participants with their dominant upper extremity. However, the comparisons of performance between the ipsi- and contralesional arm yielded inconsistent results. The novel risk metric showed that participants after stroke exhibited riskier interactions with their ipsilesional upper extremity, but did not distinguish from able-bodied performance. Lastly, mutual information, which quantified predictability between the cup and ball dynamics, showed reliable differences between the ipsilesional and contralesional upper extremity; both differed from the dominant upper extremity of able-bodied participants. It also differentiated between performance in the two difficulty levels.

With exception of risk, all metrics identified more homogeneity within individual subjects and differentiated between severity levels. However, mutual information showed the most consistent differences between PS subjects, demonstrating its potential use for quantifying severity. These first results validated the MAGIC Table as a testing device and that quantification of essential differences between individuals is possible, especially with the novel metric of predictability. This study sets the stage for extensive testing of a larger cohort of persons with stroke. Future work will further evaluate the robustness of quantification of impairment severity and recovery in the context of functional skill.

### A. Assessing Persons With Stroke in a Functional Task

To date, clinical scales have evaluated impairment in isolated postures and in movements that do not reflect functional actions in real life that are central to independent living, such as eating with a spoon, or pouring water from a bottle. The challenges that these interactive skills pose to coordination go far beyond those of simple reaching movements that have been widely studied in fundamental movement neuroscience [22], [34]. When carrying a cup with sloshing liquid inside, the person has to employ sophisticated control strategies as the fluid acts on the cup and can perturb the hand. The cup-and-ball paradigm was developed by Sternad and colleagues to shed light on such interactive tasks. A series of experimental and theoretical studies revealed that humans make interactions more predictable by choosing appropriate initial conditions, seeking stable regions in state space, and exploiting resonance frequencies [9], [10], [11], [15]. The MAGIC Table represents a real-world version of the same task that allows for affordable testing outside the laboratory. Following earlier pilot tests in children with dystonia [19], this study examined functional skill in persons with stroke. Results demonstrated that persons with moderate impairments can perform the experimental task showing that the MAGIC Table can leverage fundamental insights to gain deeper understanding of upper extremity deficits after stroke [44].

### B. Sensitivity and Versatility of the MAGIC Table for Evaluation and Rehabilitation

Traditional assessments of functional skill after stroke, such as the Wolf Motor Assessment Test, do not record kinematics, leaving evaluation to rater scores [6], [7]. These scales have limited resolution and often result in scores that suffer from ceiling or floor effects. The high-resolution kinematic recordings of the MAGIC Table not only allow quantitative assessment of performance, but also afford adjustments of task difficulty to include individuals with a range of motor ability. In addition to customizing cup parameters, task difficulty can also be manipulated by changing ball size and weight. The movement can consist of different paths as the white board allows drawing other targets. The light-weight construction of the MAGIC Table also affords use at the bedside during acute stroke hospitalization. Thus, the MAGIC Table can be used in different clinical settings, ranging from acute care hospitals to rehabilitation facilities to outpatient clinics.

In traditional rehabilitation, therapeutic exercises consist of upper extremity tasks, strength training and motor games. Improvements have largely remained task-specific with limited potential to translate to activities of daily living (ADLs) [1]. Patients also struggle to gain access to clinician-administered therapy due to insurance restrictions, geographical distance, or transportation costs. Yet, therapy is most impactful when there are a high number of repetitions coupled with sufficient motivation [35], [36]. The design features of the MAGIC Table directly address these desiderata: The portable low-cost testbed can be used at home, limiting cost and travel. The task set-up and execution with data collection require very little training and can be conducted by a clinician, caretaker, or by the user. Data could also be recorded and monitored remotely by a physical therapist. The MAGIC Table’s versatility may encourage a sufficient amount of practice without loss of motivation, which is essential for adherence to therapy.

### C. Sensitive Evaluation of Skill in a Functional Task

Movement after stroke is often slow with increased intermittency and its overall duration has been shown to correlate with functional scores [21], [37]. However, this experiment, with a more demanding task goal, showed that movement time also depended on task difficulty. Movement times significantly increased from the easiest task (with an empty cup without any additional dynamics) to the two more difficult variations where the ball acted on the cup. This indicates that more challenging tasks may better reveal motor deficits, even in straightforward kinematic metrics such as movement time.

It has been amply demonstrated that goal-directed upper limb movements in persons with stroke exhibit temporal latencies and spatial intermittencies that result in decreased smoothness, typically quantified by mean jerk [21], [23], [37], [38], [39]. However, these previous results were obtained in free reaches or in translation of rigid objects, which only increase the mass of the arm, but do not involve additional dynamics. In the present experiment, kinematics were more smooth once the ball was added to the cup, indicating that as objects with internal dynamics posed more difficulty, they required different control strategies. While smoothness differentiated between participants after stroke and able-bodied participants, it could not distinguish between the two upper extremities after stroke. One possible reason is that estimates of smoothness cannot differentiate between the participants’ inherent intermittency and the additional task dynamics.

Previous research on the virtual cup-and-ball task showed that healthy older participants initially moved with higher risk of spilling the ball but learned to decrease risk values, when unconstrained by time [30]. In this study subjects were asked to move the cup-and-ball as fast as possible. Unexpectedly, risk in the contralesional upper extremity was significantly lower than in the ipsilesional upper extremity, although the trend was largely driven by two participants (PS4 and PS5). Nevertheless, this result may be due to compensatory strategies that individuals may have developed with their ipsilesional upper extremity. Participants with apparent arm weakness appear to engage in less risky strategies. This is likely due to their slower movements, where less force is applied to the ball, reducing likelihood of escape. Hence, low risk values coupled with slow movements may reflect that persons with stroke were unable to retain control of the ball at faster cup speeds.

Previous work by Sternad and colleagues demonstrated that humans seek to increase predictability when interacting with objects that have potentially unpredictable dynamics [9], [10], [18]. Predictability of object dynamics was quantified by an information-theoretic metric, mutual information, conceptually similar to correlation, where higher *MI* expresses a greater degree of predictability. Not only was the degree of predictability attained in able-bodied participants higher than participants after stroke, but also interaction with the ipsilesional side was more predictable than that of the contralesional side. Further, the ICC scores demonstrated a high within-person consistency of this metric. Whether this metric aligns with clinical scores or may be even more discerning in diagnosing deficits remains to be verified in future work. Quantifying predictability in functional interactions may yield subtle insights that are otherwise missed by conventional scales.

### D. Limitations

The MAGIC Table was designed to be inclusive to individuals with a range of motor abilities. Yet, those with moderate and severe upper extremity deficits may be unable to grasp the cup handle, requiring their hand to be secured with a loose bandage to the cup. Further issues may arise when patients are unable to extend their arm without compensation by the torso. Support of the upper arm against gravity may help to extend their reach or improve quality of the trajectories, but it has also been shown to result in less improvements in reach radius across practice [40], [41], [42]. This is consistent with results in healthy individuals, where arm weight support was also shown to hinder learning of an object manipulation task [43]. To make the testbed more inclusive, we will evaluate the effectiveness of arm support and a grip glove in future experiments.

Future studies should also add systematic monitoring and clinical assessments, such as Fugl-Meyer testing synchronous with the experimental data collection. However, this may also create discrepancies between improvements seen in scores and MAGIC Table results that will need further scrutiny to decide which results are more reliable. While the extension-flexion movements of the cup-and-ball were designed to make the task as inclusive to various skill levels, moving the cup along a line reduced the dimensionality of the task. Future experiments should examine tasks that include two-dimensional movements, as they make the cup-ball interactions more complex and demanding, possibly becoming even more sensitive testbeds. This is feasible given that the cup-and-ball can be moved across the whiteboard and the computer vision algorithm can detect the ball and the cup at any point within the frame of view.

## V. CONCLUSION

This study demonstrated that the MAGIC Table can be used as a portable low-cost device for quantitative assessment of upper-extremity movements after stroke. Inspired by daily motor challenges that can be manipulated for experimental purposes, the paradigm is grounded in theoretical analysis and presents a bridge between laboratory research and clinical field data. The findings highlight the sensitivity of the novel measures to become useful in patient evaluation. With its feasibility and affordable design, the MAGIC Table has significant potential to be an informative rehabilitation assessment device.

## Figures and Tables

**TABLE I:** PARTICIPANT DEMOGRAPHICS

Participant	Age	Gender	Dominant Side	Contralesional Side	National Institutes of Health Stroke Scale Left Arm Sub-item	National Institutes of Health Stroke Scale Right Arm Sub-item	Fugl-Meyer Score	Days Since Stroke Event at Session
**PS1**	86	Female	Right	Left	1	0	40	11
**PS2**	56	Male	Right	Left	1	0		217
**PS3**	67	Male	Right	Right	0	1	43	209
**PS4**	57	Male	Right	Right	0	0		108
**PS5**	53	Female	Right	Left	1	0	50	100
**PS6**	39	Male	Left	Right	0	0		682
**AP1**	69	Male	Right					
**AP2**	71	Female	Right					
**AP3**	67	Female	Right					
**AP4**	44	Male	Right					
**AP5**	61	Male	Right					
**AP6**	60	Female	Right					
